# The Work of the Digestive Glands

**Published:** 1913-03

**Authors:** 


**The Work of the Digestive Glands.** By Professor I. P. PAVLOV. Translated by W. H. THOMPSON. Pp. xiv, 255. London: Charles Griffin & Co. 1910. Price, 10s. 6d. net,

The appearance of the first edition of this work was welcomed not only by the physiologists, but also by the practical clinicians, for the experiments of Pavlov and his assistants explained much that had previously beem empirical, and reduced the number of the "off-chance" prescriptions and dietaries which are given to patients suffering from disorders of digestion.

The new edition, we observe, still gives on the title-page Professor Pavlov as author and Professor Thompson as translator, an arrangement that hardly gives an accurate version of their relative responsibilities.

Professor W. H. Thompson has very greatly increased for clinicians the value of the original book by adding two chapters of his own on the muscular movements of the alimentary canal. Since the X-rays have made the investigation of these movements comparatively easy, it has become at least as necessary to be familiar with the normal movements as it is to be familiar with the physiology of the digestive juices of the alimentary canal.

Recent work has necessitated the re-writing of a whole chapter, and various additions have been made throughout the book.

Those who wish to keep abreast with modern theory as it affects practice must obtain this book.

